# Prioritized Task Distribution Considering Opportunistic Fog Computing Nodes

**DOI:** 10.3390/s21082635

**Published:** 2021-04-09

**Authors:** Yeunwoong Kyung

**Affiliations:** School of Computer Engineering, Hanshin University, Osan 18101, Korea; ywkyung@hs.ac.kr

**Keywords:** fog computing, opportunistic fog, task distribution

## Abstract

As service latency and core network load relates to performance issues in the conventional cloud-based computing environment, the fog computing system has gained a lot of interest. However, since the load can be concentrated on specific fog computing nodes because of spatial and temporal service characteristics, performance degradation can occur, resulting in quality of service (QoS) degradation, especially for delay-sensitive services. Therefore, this paper proposes a prioritized task distribution scheme, which considers static as well as opportunistic fog computing nodes according to their mobility feature. Based on the requirements of offloaded tasks, the proposed scheme supports delay sensitive task processing at the static fog node and delay in-sensitive tasks by means of opportunistic fog nodes for task distribution. To assess the performance of the proposed scheme, we develop an analytic model for the service response delay. Extensive simulation results are given to validate the analytic model and to show the performance of the proposed scheme, compared to the conventional schemes in terms of service response delay and outage probability.

## 1. Introduction

In the modern world, lots of smart devices, including smartphones, wearable devices, factory facilities, and vehicles, have been equipped with various sensors and are connected between devices. This realizes the internet of things (IoT) networks, where information is collected and shared among the connected devices and also accelerates the commercialization of IoT applications, such as smart factory, smart home, and smart environment [1,2].

Since IoT applications have their own service requirements according to their characteristics, each service should be processed and provided to guarantee the requirements. For example, video-on-demand (VoD), online gaming, augmented reality (AR), and virtual reality (VR) are typically delay-sensitive services that need low latency. On the other hand, a large portion of data traffic can tolerate relatively long latency, such as pushing of contents to the edge, log and backup services, and tenant delivery [3,4,5]. According to the various service requirements, efficient resource utilization to process the requests of services should be considered.

Since IoT devices have limited resources in terms of computing and energy, the cloud service has been provided to process the data generated from lots of IoT devices owing to the flexible utilization of computing resources and support of high volume with fast scalability [6]. However, the physical distance between the cloud servers and IoT devices results in long latency and consumes high bandwidth of the core network. In addition, the load can be concentrated to the cloud according to the number of IoT devices. In order to address these challenges, the concept of fog computing is introduced, where computing resources are moved close to the IoT devices to distribute the load of the cloud, minimize latency for IoT services, and reduce core network resource usage [7,8].

In the fog computing environment, the requests of IoT devices usually can be offloaded to the fog computing nodes (FNs) co-located with the access point (AP) such as the base station (BS) where IoT devices are connected [9,10]. This physical proximity can provide low latency. However, a bottleneck can occur when the number of requests of IoT devices increase. To handle this problem, there have been works on the task distribution of IoT devices considering the co-work with other FNs and collaboration between the cloud and FNs [9,11,12,13,14], as well as context awareness [15,16].

Recently, task distribution considering not only static FNs but also mobile FNs has gained increasing attention [17,18,19,20,21,22,23]. Owing to the intermittent availability of mobile FNs, they are considered as the concept of opportunistic FNs (OFNs) [18]. Research coverage on OFNs has been extended to include smart phones, vehicles, and unmanned aerial vehicles (UAVs) [21].

As mentioned above, OFNs are not always available because of mobility and intermittent connectivity. In addition, communication and computing latency can be additionally considered. This means that the service requirements of delay-sensitive services cannot be guaranteed through offloading with the OFNs [18]. In order to handle this problem, this paper introduces the prioritized task distribution scheme, which utilizes OFNs when they are available only for delay-tolerant tasks and static FNs for delay-sensitive tasks. To evaluate the performance of the proposed scheme, we develop the analytical model for response delay. Extensive simulation results validate the analytic model and demonstrate that the proposed scheme has lower response delay while maintaining the outage probability at a low level compared to that of the conventional schemes.

The key contribution of this paper is two-fold: (1) this paper develops an analytic model based on Markov chain of the proposed and conventional schemes for the response delay; and (2) by means of extensive simulation works, this paper demonstrates the performance of the proposed and conventional schemes under various environments, which can be a valuable design reference for OFN-based architecture.

The remainder of this paper is organized as follows. After related work is reviewed in Section 2, system models for the proposed and conventional schemes are given in Section 3. Simulation results and concluding remarks are described in Section 4 and Section 5, respectively.

## 2. Related Work

In cloud and fog architecture, the task distribution of IoT devices has been discussed a lot [9,11,12,13,14,15,16]. Task distribution schemes were provided by associating tasks into suitable FNs to minimize the service response delay considering communications and processing procedures [9,11]. In addition, collaborative computing between cloud and fog (i.e., load sharing) was utilized to achieve better delay performance by means of optimal task splitting [12,13]. Yi et al. [14] presented a different role between fog and cloud. For example, local and regional task can be processed on fog to provide timely feedback, such as emergency cases and computational-intensive task can be scheduled on the cloud. Kayes et al. [15] reviewed the previous context aware access control approaches and provided general requirements with challenging issues to provide context-awareness of the fog-based access control. Moreover, a fog-based, context-aware access control scheme was proposed [16], which provides the benefits of a unified data model and its associated access and privacy control policies to reduce the administrative and processing overheads. Although these works did not consider the OFNs, their efforts became the groundwork for works on OFN-based task distribution.

There have been lots of studies on distribution of load utilizing OFNs with different objectives [17,18,19,20,21,22,23]. Minimizing service latency has been one of the major issues [17,22]. A dynamic task allocation scheme was proposed utilizing both static FN and OFN to optimize the service latency and quality loss rate [17]. Although service interruption due to the mobility of OFN was mentioned, it does not consider the differentiation of delay-sensitive flows, which can result in a long response delay for flows due to the repetitive resource re-allocation. Wang et al. [22] introduced a model with parked and moving vehicles (i.e., FNs and OFNs) to minimize the average system response time. Since it also allocates the request to OFNs without differentiation, the performance requirements of delay-sensitive services cannot be guaranteed due to the mobility of OFNs even though the average performance can be improved. In addition, various issues on OFN have been covered as follows. Fernando et al. [18] reviewed motivations and identified the requirements to enable OFN applications. Then, they provided a model of OFNs to support IoT applications, especially for hazardous and volatile events. Ning et al. [19] introduced an energy-efficient scheduling scheme. It schedules the task to the static FN and OFN in a cooperative manner to minimize the energy consumption of network access devices within the delay constraint. Liu et al. [20] formulated a task scheduling decision problem based on task dependency requirements to reduce the average completion time. Zhou et al. [21] investigated a computation resource allocation problem for the task assignment to optimize long-term network delay performance. To motivate OFNs for resource sharing, they utilized a contract-based incentive mechanism. Liu et al. [23] analyzed the utility-based task distribution model according to the mobility of OFNs. They focused on the temporal and spatial characteristics of the relationships between OFNs without considerations on FNs.

Unlike the aforementioned works, this paper mainly considers the differentiation of service requests and distribution according to the availability of OFN.

## 3. System Model

### 3.1. System Architecture

**Figure 1:**
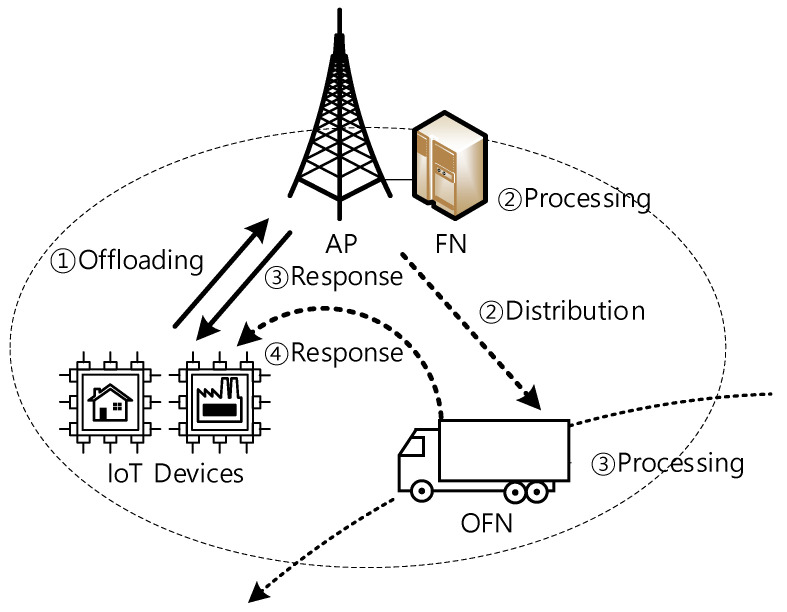
System architecture.

Figure 1 depicts the system architecture, where IoT devices offload tasks to FN and wait for a response. As shown in Figure 1, the offloading can be processed by either OFN or FN when OFN is available (or not) due to mobility. This paper assumes that OFN notifies its events of entering and leaving and reports current computing status to AP, based on the existing cellular registration mechanism [17].

As mentioned above, since there are various requirements, depending on the IoT applications, this paper divides request flows into high priority (HP) flows (which require delay constraint) and low priority (LP) flows (which are delay tolerant). The criteria of this differentiation can be changed according to the network status and operator’s policy.

Offloading to OFN enables the load distribution of FN. This means the response of FN can be reduced. However, flows processed by OFN can have longer response delay than that of FN because the offloading to OFN needs an additional delivery procedure and the computing resource of OFN cannot always be guaranteed.

Therefore, load distribution should be performed considering the flow differentiation and features of FN and OFN, as described above.

### 3.2. System Model of the Proposed Scheme

**Figure 2:**
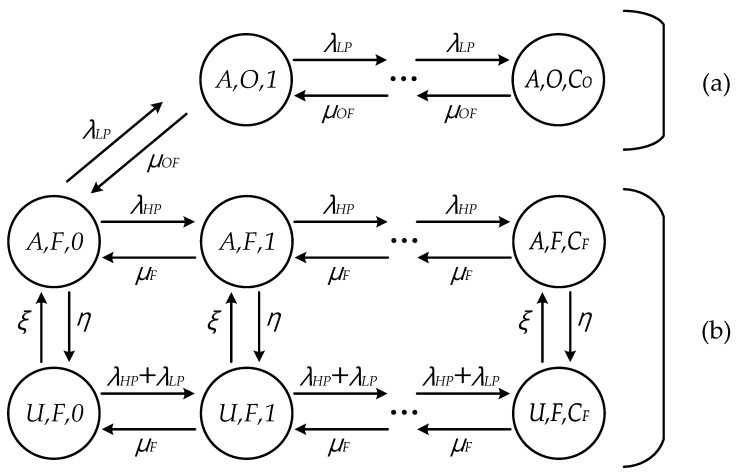
Markov chain model of the proposed scheme: (**a**) OFN; (**b**) FN.

To develop an analytical model, this paper considers M/M/1 queuing models for fog computing architecture [10,17], where the request flows of HP and LP follow Poisson distribution with rates *λ_HP_* and *λ_LP_*. In addition, since the task sizes of the request flows are assumed to follow the exponential distribution, the service times of FN and OFN also follow the exponential distribution with mean of 1/*μ_F_* and 1/*μ_OF_*, respectively [9]. Figure 2 shows the Markov chain model of FN and OFN in the proposed scheme. In state (*i*, *n*, *j*), *i* represents the status of availability for OFN, *n* means the serving node to process the request, and *j* denotes the number of requests currently served by *n*. Each request can be served by either FN (*F*) or OFN (*O*), where *C_F_* and *C_O_* are the capacities of FN and OFN, respectively. Note that each FN (OFN) can have different values of *C_F_* (*C_O_*) depending on its own capability. Status *A* means that the offloading to OFN is available because it is located in the coverage of AP. On the other hand, OFN is unavailable at status *U*. The sojourn time of OFN at status *A* and *U* follows the exponential distribution with rates 1/*η* and 1/*ξ*, respectively [23,24,25]. In Figure 2, LP flows are only be offloaded to OFN at status *A;* to distributed the load of FN and HP, flows are processed by FN to reduce the latency. At status *U*, FN processes both HP and LP in a round-robin fashion because OFN is not available. This paper assumes that the requests from IoT devices within the range of AP are offloaded to FN or OFN connected directly with AP. Collaborative offloading with other APs and cloud [9,11] and prioritized processing even at status *U,* such as using priority queue allocation [26], will be one of our future works.

The transition rates of FN in Figure 2 can be obtained as follows.

(1) $$\begin{array}{cc} p(A,F,j;U,F,j)=\eta & \left(0\leq j\leq C_{F}\right)\\ p(U,F,j;A,F,j)=\xi & \left(0\leq j\leq C_{F}\right)\\ p(A,F,j;A,F,j-1)=\mu_{F} & \left(0\leq j\leq C_{F}\right)\\ p(A,F,j;A,F,j+1)=\lambda_{HP} & \left(0\leq j\leq C_{F}\right)\\ p(U,F,j;U,F,j-1)=\mu_{F} & \left(0\leq j\leq C_{F}\right)\\ p(U,F,j;U,F,j+1)=\lambda_{HP}+\lambda_{LP} & \left(0\leq j\leq C_{F}\right)\\ p(A,F,j;A,O,j+1)=\lambda_{LP} & \left(j=0\right)\\ p(A,O,j;A,O,j+1)=\lambda_{LP} & \left(1\leq j\leq C_{O}\right)\\ p(A,O,j;A,O,j-1)=\mu_{OF} & \left(1\leq j\leq C_{O}\right)\\ p(A,F,j;A,O,j+1)=\lambda_{LP} & \left(j=1\right)\\ p(A,O,j;A,F,j-1)=\mu_{OF} & \left(j=1\right) \end{array}$$

In order to find out steady state probability *π_i,n,j_*, the balance equations can be calculated as follows:

(2) $$\begin{array}{l} (1)i=A,n=F,j=0,(\lambda_{HP}+\lambda_{LP})\pi_{i,n,j}=\mu_{F}\pi_{i,n,j+1}+\xi \pi_{U,n,j}+\mu_{OF}\pi_{i,O,j+1}\\ (2)i=A,n=F,0<j<C_{F},(\lambda_{HP}+\eta +\mu_{F})\pi_{i,n,j}=\lambda_{HP}\pi_{i,n,j-1}+\mu_{F}\pi_{i,n,j+1}+\xi \pi_{U,n,j}\\ (3)i=A,n=F,j=C_{F},(\eta +\mu_{F})\pi_{i,n,j}=\lambda_{HP}\pi_{i,n,j-1}+\xi \pi_{U,n,j}\\ (4)i=U,n=F,j=0,(\xi +\lambda_{HP}+\lambda_{LP})\pi_{i,n,j}=\mu_{F}\pi_{i,n,j+1}+\eta \pi_{A,n,j}\\ (5)i=U,n=F,0<j<C_{F},(\xi +\lambda_{HP}+\lambda_{LP}+\mu_{F})\pi_{i,n,j}=(\lambda_{HP}+\lambda_{LP})\pi_{i,n,j-1}+\mu_{F}\pi_{i,n,j+1}+\eta \pi_{A,n,j}\\ (6)i=U,n=F,j=C_{F},(\xi +\mu_{F})\pi_{i,n,j}=(\lambda_{HP}+\lambda_{LP})\pi_{i,n,j-1}+\eta \pi_{A,n,j}\\ (7)i=A,n=O,j=1,(\mu_{OF}+\lambda_{LP})\pi_{i,n,j}=\mu_{OF}\pi_{i,n,j+1}+\lambda_{LP}\pi_{U,F,j-1}\\ (8)i=A,n=O,1<j<C_{OF},(\lambda_{LP}+\mu_{OF})\pi_{i,n,j}=(\lambda_{LP})\pi_{i,n,j-1}+\mu_{OF}\pi_{i,n,j+1} \end{array}$$

Because of the complexity of closed-forms for *π_i,n,j_*, this paper utilizes an iterative algorithm to obtain *π_i,n,j_* [26]. To get the response delay of HP flow, the average number of HP requests (*N_H_*) in FN can be given by:

(3) $$N_{H}=\sum_{i=A,U}\sum_{j=0}^{C_{F}}j\pi_{i,F,j}$$

The average number of LP requests (*N_L_*) can also be given by:

(4) $$N_{L}=\sum_{j=0}^{C_{F}}j\pi_{U,O,j}+\sum_{j=1}^{C_{O}}j\pi_{A,O,j}$$

In addition, by considering each status of FN, the effective request arrival rate of HP requests (*λ_eH_*) can be calculated as follows:

(5) $$\lambda_{eH}=\sum_{j=0}^{C_{F}}\lambda_{HP}\pi_{A,F,j}+\sum_{j=0}^{C_{F}}(\lambda_{HP}+\lambda_{LP})\pi_{U,F,j}$$

In the same way, the effective request arrival rate of LP requests (*λ_eL_*) can be calculated as follows:

(6) $$\lambda_{eL}=\sum_{j=1}^{C_{O}}\lambda_{LP}\pi_{A,O,j}+\sum_{j=0}^{C_{F}}(\lambda_{HP}+\lambda_{LP})\pi_{U,F,j}$$

Then, by means of Little’s law [27], the average response delay of HP (*W_HP_*) and LP (*W_LP_*) flows can be obtained by (7) and (8), respectively:

(7) $$W_{HP}=\frac{N_{H}}{\lambda_{eH}}$$

(8) $$W_{LP}=\frac{N_{L}}{\lambda_{eL}}$$

Even though the proposed scheme preferentially handles the HP flow, the response delay cannot guarantee the required delay constraint if the amount of incoming requests increases continuously or the incoming requests are concentrated instantly. Therefore, the outage probability to show the QoS degradation, wherein the request cannot get a response with application delay constraint, will be analyzed in the next chapter.

On the other hand, if the delay constraint is always guaranteed when the amount of incoming request is small, the distribution to the OFN can be preferred or not based on the network policy. Even in this situation, this paper utilizes the OFN as shown in Figure 2 because making the best use of OFN is efficient for scalability [28].

### 3.3. System Model of the Conventional Scheme

**Figure 3:**
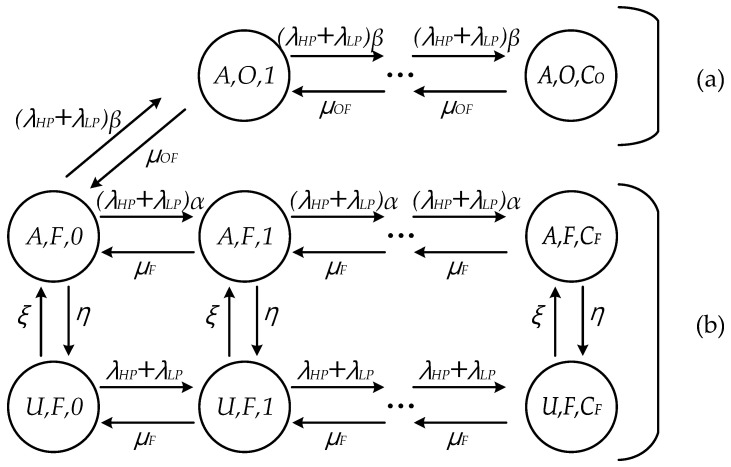
Markov chain model of the conventional scheme: (**a**) OFN; (**b**) FN.

Compared to the proposed scheme where incoming requests are classified into HP and LP flows and processed based on this classification as shown in Figure 2, the conventional scheme offloads the incoming request to FN and OFN without flow differentiation to make the best use of available resources [21]. This means all the incoming requests can be processed evenly by both FN and OFN if OFN is available or by only FN if OFN is unavailable, as shown in Figure 3. Especially when OFN is available, since the incoming requests can be distributed to FN and OFN, the proportions of the incoming requests to FN and OFN are set to *α* and *β*, respectively (i.e., *α* + *β* = 1). Since the differentiation is not considered in the conventional scheme, the response delay for the incoming requests depends on the proportions (*α, β*), irrespective of the delay requirement.

The transition rates of FN in Figure 3 can be obtained as follows:

(9) $$\begin{array}{cc} p(A,F,j;U,F,j)=\eta & \left(0\leq j\leq C_{F}\right)\\ p(U,F,j;A,F,j)=\xi & \left(0\leq j\leq C_{F}\right)\\ p(A,F,j;A,F,j-1)=\mu_{F} & \left(0\leq j\leq C_{F}\right)\\ p(A,F,j;A,F,j+1)=(\lambda_{HP}+\lambda_{LP})\alpha & \left(0\leq j\leq C_{F}\right)\\ p(U,F,j;U,F,j-1)=\mu_{F} & \left(0\leq j\leq C_{F}\right)\\ p(U,F,j;U,F,j+1)=\lambda_{HP}+\lambda_{LP} & \left(0\leq j\leq C_{F}\right)\\ p(A,F,j;A,O,j+1)=(\lambda_{HP}+\lambda_{LP})\beta & \left(j=0\right)\\ p(A,O,j;A,F,j-1)=\mu_{OF} & \left(j=1\right)\\ p(A,O,j;A,O,j+1)=(\lambda_{HP}+\lambda_{LP})\beta & \left(1\leq j\leq C_{O}\right)\\ p(A,O,j;A,O,j-1)=\mu_{OF} & \left(1\leq j\leq C_{O}\right) \end{array}$$

The difference between Equations (1) and (6) is the transition probability from state *j* to *j* + 1 at status *A*. In order to find out steady state probability *π_i,n,j_*, the balance equations can be calculated as follows:

(10) $$\begin{array}{l} (1)i=A,n=F,j=0,(\lambda_{HP}+\lambda_{LP})\pi_{i,n,j}=\mu_{F}\pi_{i,n,j+1}+\xi \pi_{U,n,j}+\mu_{OF}\pi_{i,O,j+1}\\ (2)i=A,n=F,0<j<C_{F},(\lambda_{HP}+\lambda_{LP}+\eta +\mu_{F})\pi_{i,n,j}=(\lambda_{HP}+\lambda_{LP})\pi_{i,n,j-1}+\mu_{F}\pi_{i,n,j+1}+\xi \pi_{U,n,j}\\ (3)i=A,n=F,j=C_{F},(\eta +\mu_{F})\pi_{i,n,j}=(\lambda_{HP}+\lambda_{LP})\pi_{i,n,j-1}+\xi \pi_{U,n,j}\\ (4)i=U,n=F,j=0,(\xi +\lambda_{HP}+\lambda_{LP})\pi_{i,n,j}=\mu_{F}\pi_{i,n,j+1}+\eta \pi_{A,n,j}\\ (5)i=U,n=F,0<j<C_{F},(\xi +\lambda_{HP}+\lambda_{LP}+\mu_{F})\pi_{i,n,j}=(\lambda_{HP}+\lambda_{LP})\pi_{i,n,j-1}+\mu_{F}\pi_{i,n,j+1}+\eta \pi_{A,n,j}\\ (6)i=U,n=F,j=C_{F},(\xi +\mu_{F})\pi_{i,n,j}=(\lambda_{HP}+\lambda_{LP})\pi_{i,n,j-1}+\eta \pi_{A,n,j}\\ (7)i=A,n=O,j=1,\beta (\lambda_{HP}+\lambda_{LP})\pi_{i,n,j}=\mu_{OF}\pi_{i,n,j+1}+\beta (\lambda_{HP}+\lambda_{LP})\pi_{i,F,j-1}\\ (8)i=A,n=O,0<j<C_{O},\beta (\lambda_{HP}+\lambda_{LP})\pi_{i,n,j}=\beta (\lambda_{HP}+\lambda_{LP})\pi_{i,n,j-1}+\mu_{OF}\pi_{i,n,j+1} \end{array}$$

As mentioned above, *π_i,n,j_* can be obtained using an iterative algorithm. In addition, by means of Equations from (3) to (8), the average response delay of HP and LP flows can also be calculated.

In addition, the basic scenario without OFN can be modelled only considering status *A* in Figure 3.

## 4. Performance Analysis

In this section, we evaluate the performance of the proposed scheme compared with the conventional scheme without differentiation (NoDiff) [21] and basic scenario without OFN. For numerical analysis, the average service time of FN is assumed to be 1 ms. Since OFNs generally have limited capacity compared to FNs [29], this paper assumes that the average size of the total capacity of FN (*C_F_*) and OFN (*C_O_*) is set as 20 and 10, respectively. For NoDiff, the requests are evenly distributed to FN and OFN by AP when OFN is available (i.e., both *α* and *β* are set to 1/2). To verify the analytical results marked as (A), event-driven simulations based on MATLAB R2018a are conducted and the simulation results are marked as (S) in the following figures. In the simulations, this paper assumes that *C_F_* and *C_O_* follow uniform distribution from 17 to 23 and from 7 to 13, respectively. Arrival times of events with *C_F_* and *C_O_* are drawn by generating 50,000 random numbers according to the distribution, and then the response delay and outage probability are computed.

### 4.1. Response Delay

**Figure 4:**
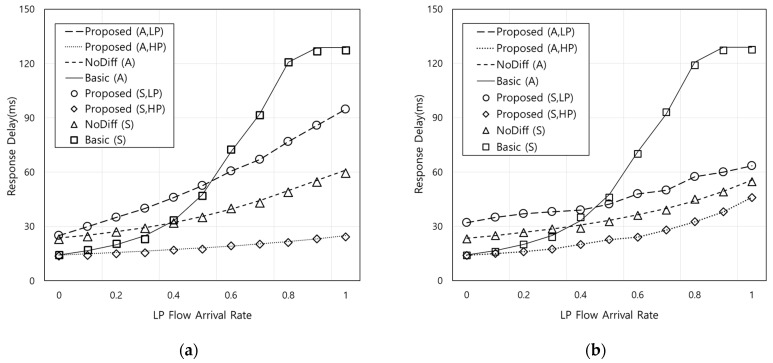
Response delay according to the LP flow arrival rate (A: analytic results, S: simulation results): (**a**) *η* = 1/3, *ξ* = 1/3, (**b**) *η* = 2/3, *ξ* = 1/3.

Figure 4 shows the response delay according to the LP flow arrival rate when the HP flow arrival rate is 0.3. Both *η* and *ξ* are set to 1/3 in Figure 4a and *η* and *ξ* are 2/3 and 1/3, respectively, in Figure 4b. First of all, as shown in Figure 4, simulation results are almost consistent with analytic results in all simulation settings. In Figure 4a, the NoDiff scheme has a higher response delay compared to that of HP flows in the proposed scheme because it makes the best use of FN and OFN without the differentiation when OFN is available to be utilized. In addition, the response delay of NoDiff scheme is higher than that of the basic scheme when LP flow arrival rate is low. This is because NoDiff offloads the incoming request to OFN, which has relatively low capacity when OFN is available even though the load of FN is not high. Note that this effect can be higher if the latency between FN and OFN is considered, although they are not included in this paper. Therefore, it can be noticed that it is required to differentiate the requests for the delay performance with efficient available resource utilization. For example, the response delay of the proposed scheme is about 49% and 62% shorter than that of NoDiff and the basic scheme, respectively, when LP flow arrival rate is 0.5 (i.e., fifth *x*-axis point in Figure 4a). On the other hand, LP flows in the proposed scheme have a higher response delay compared to those of NoDiff because LP flows are processed only using OFNs when OFNs are available. Figure 4b shows a similar trend with Figure 4a. However, the difference of the response delay between the proposed and NoDiff schemes does not increase according to the LP flow arrival rate, compared to that in Figure 4a. This is because the period when FN processes all the requests by itself increases owing to the lower sojourn time than that of Figure 4a. Comparing Figure 4b with Figure 4a, the response delay of LP flows becomes improved while HP flows of the proposed scheme has the lowest response delay among all schemes. From the results, in order to prevent LP flows from starvation, the appropriate criteria for the differentiation between HP and LP flows should be determined based on the network status such as OFN sojourn time, delay constraint, and the amount of incoming requests. The optimal solution to find the criteria will be one of our future works.

**Figure 5:**
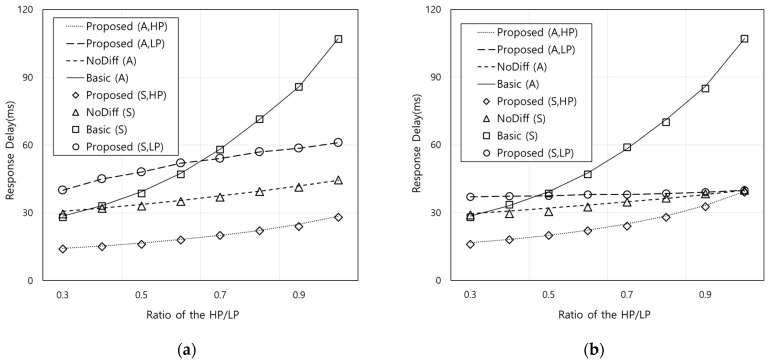
Response delay according to the ratio of HP flow arrival rate to LP flow arrival rate (A: analytic results, S: simulation results): (**a**) *η* = 1/3, *ξ* = 1/3, (**b**) *η* = 2/3, *ξ* = 1/3.

Figure 5 shows the response delay according to the ratio of HP flow arrival rate to LP flow arrival rate when the LP flow arrival rate is 0.5. Both *η* and *ξ* are set to 1/3 in Figure 5a and *η* and *ξ* are 2/3 and 1/3, respectively in Figure 5b. Figure 5 also shows similar trend with Figure 4 because the proposed scheme performs differentiated processing of HP flow requests. In Figure 5b, it can be noted that the effects of the proposed scheme can be reduced according to the ratio of HP and LP flow arrival rate. This also means that the impact of the differentiation between HP and LP flows becomes smaller. For example, the differences in response delay for the HP flows in the proposed scheme from that of NoDiff and LP flows in the proposed scheme are 12.9 ms and 21.5 ms when the ratio is 0.3, and 0.6 ms and 0.9 ms when the ratio is 1, respectively. This is because in the proposed scheme, the amount of HP flow requests to FN increases under the capacity constraint and the amount of LP flow requests processed by OFN is reduced owing to the lower sojourn time than that of Figure 5a.

### 4.2. Outage Probability

**Figure 6:**
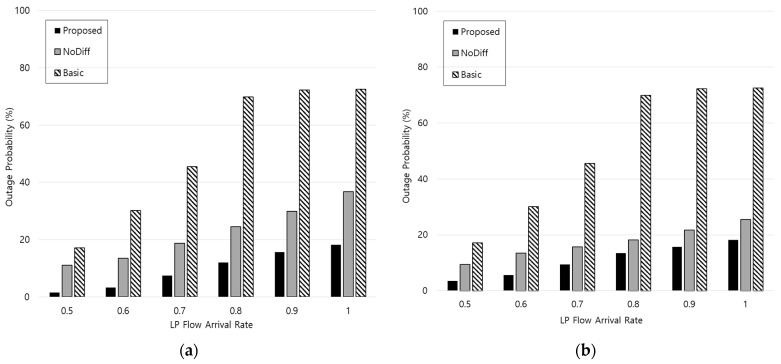
Outage probability of HP flow requests: (**a**) *η* = 1/3, *ξ* = 1/3, (**b**) *η* = 2/3, *ξ* = 1/3.

Figure 6 shows the outage probability (i.e., the ratio of the number of HP flow requests which do not satisfy the delay constraints to the total number of HP flow requests). Both *η* and *ξ* are set to 1/3 in Figure 6a and *η* and *ξ* are 2/3 and 1/3, respectively in Figure 6b. The delay constraint is assumed to be 30 ms. From Figure 6, outage probability increases according to the LP flow arrival rate because the system has a capacity constraint. However, the proposed scheme can have lower outage probability, which means a higher QoS satisfaction ratio compared to the conventional schemes because it preferentially handles the HP flow requests. As explained above, since the response delay of the proposed scheme becomes higher with increasing *η*, the outage probability also increases when comparing Figure 6a with Figure 6b. However, the proposed scheme still has lower outage probability compared to the conventional schemes because of the flow differentiation. Maintaining the outage probability below the specific value is an important performance metric from a network operator’s perspective.

## 5. Conclusions

In this paper, a prioritized task distribution scheme considering opportunistic fog computing nodes in the fog computing environment is proposed. The proposed scheme differentiates incoming flow requests into delay-sensitive and delay-insensitive flows. Then, delay-sensitive flows can be processed by static FN to support the delay requirement. On the other hand, the proposed scheme makes the best use of OFN for delay-insensitive flows to reduce the load of the static FN. Numerical and simulation results show that the proposed scheme can provide lower service delay for the delay-sensitive flows, compared to the conventional schemes, while maintaining the outage probability at a low level. In our future work, experiments considering the real environment with commercial IoT devices and mobile computing node to distribute tasks will be performed.

## Data Availability

Not applicable.
